# Pancreatectomy for metastasis to the pancreas from colorectal cancer and reconstruction of superior mesenteric vein: a case report

**DOI:** 10.1186/1752-1947-5-424

**Published:** 2011-08-31

**Authors:** Efstratios Georgakarakos, Hartmut Goertz, Joerg Tessarek, Karsten Papke, Christoph Seidlmayer

**Affiliations:** 1 Department of Vascular Surgery, St Bonifatius Hospital, Wilhelmstraße 15, Lingen, Germany; 2 Department of Radiology, St Bonifatius Hospital, Wilhelmstraße 15, Lingen, Germany; 3 Department of General Surgery, St Bonifatius Hospital, Wilhelmstraße 15, Lingen, Germany

## Abstract

**Introduction:**

Tumors of the pancreatic head can infiltrate the superior mesenteric vein. In such cases, the deep veins of the lower limbs can serve as suitable autologous conduits for superior mesenteric vein reconstruction after its resection. Few data exist, however, describing the technique and the immediate patency of such reconstruction.

**Case report:**

We present the case of a 70-year-old Caucasian man with a metachronous metastasis of colon cancer and infiltration of the uncinate pancreatic process, on the anterior surface of which the tumor was located. *En bloc *resection of the tumor was performed with resection of the superior mesenteric vein and reconstruction. A 10 cm segment of the superficial femoral vein was harvested for the reconstruction. The superficial femoral vein segment was inter-positioned in an end-to-end fashion. The post-operative conduit patency was documented ultrasonographically immediately post-operatively and after a six-month period. The vein donor limb presented subtle signs of post-operative venous hypertension with edema, which was managed with compression stockings and led to significant improvement after six months.

**Conclusion:**

In cases of exploratory laparotomies with high clinical suspicion of pancreatic involvement, the potential need for vascular reconstruction dictates the preparation for leg vein harvest in advance. The superficial femoral vein provides a suitable conduit for the reconstruction of the superior mesenteric vein. This report supports the uncomplicated nature of this technique, since few data exist about this type of reconstruction.

## Introduction

Though pancreatic metastases from colorectal cancer are very rare and the mid-term results of surgery have not been clearly defined yet, pancreatic resection has been suggested in selected patients with isolated metastases from colorectal cancer and/or limited extra-pancreatic disease [1,2].

The most common unexpected finding at the time of pancreaticoduodenectomy in pancreatic carcinoma of the head and uncinate process is the invasion of the superior mesenteric vein (SMV) or superior mesenteric portal vein (SMV/PV) confluence, located anteriorly, laterally, or posterolaterally [3,4]. The current literature suggests that portal vein and/or SMV invasion is not a contraindication to pancreatic resection, provided that these veins are not occluded [5]. In this report, we describe a case of resection of the SMV and restoration of its continuity by inter-position of an autologous superficial femoral vein (SFV) graft, since few data exist about SMV reconstruction with a SFV graft during pancreatectomy.

## Case presentation

A 70-year-old Caucasian man with a history of right hemi-colectomy one year earlier (due to adenocarcinoma of the right colon) was admitted to our hospital with abdominal pain and unexplained weight loss. His laboratory values, X-rays, and computed tomography (CT) were not indicative of any distinctive pathology. Therefore, the general surgeons decided to proceed with an exploratory laparotomy, based on the patient's recent hemi-colectomy and the high clinical suspicion of a metachronous metastatic insult of the pancreas.

A metastatic tumor was identified in the uncinate process of the pancreas. During the dissection and preparation, the SMV involvement was identified on its anterior surface superiorly to the confluence of the middle colic vein at the level of the transverse mesocolon. No involvement of the superior mesenteric artery was identified. When it was decided that the tumor could be resected with a sufficient macroscopic margin, a duodenopancreatectomy was performed. Sufficient resection with healthy margins was documented by intra-operative histology. The tumor adhered only to the SMV, with the latter caudally divided at the point where the SMV emerged.

**Figure 1:**
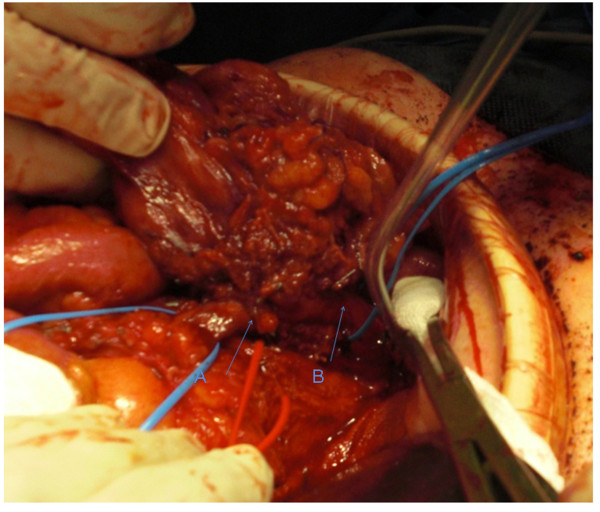
**Resection and reconstruction of the superior mesenteric vein with superficial vein segment**. **(A) **Distal anastomosis. **(B) **Proximal anastomosis. The red vessel loop encircles the superior mesenteric artery.

Accordingly, a right mid-thigh incision was performed, and an adequate SFV segment up to the junction with the profunda femoris vein was harvested. The duration from the vein preparation and harvest to skin closure was 15 to 20 minutes. During the venous reconstruction, a solution of 5000 U of heparin was delivered locally through the SMV. No valvulotomy was performed. The pancreatic head resection was immediately followed by the construction of a proximal anastomosis between a non-reversed SFV segment 3 cm to 4 cm in length and the central stump of the SMV in an end-to-end fashion (Figure 1). The peripheral anastomosis was created in a similar fashion. The duration of the creation of each anastomosis was 10 minutes. Intra-operatively, the patency of the reconstruction was confirmed by a continuous wave Doppler signal. The operation was completed with the creation of pancreatojejunostomy and a new ileotransversostomy.

**Figure 2:**
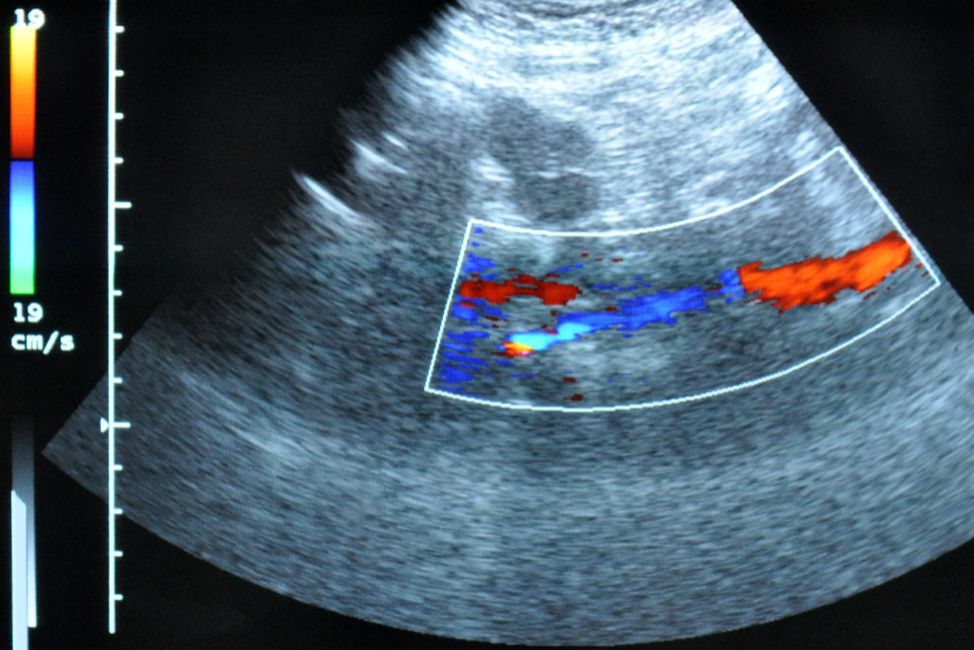
**Ultrasonographic six-month follow-up evaluation of the superficial femoral vein inter-position graft showing good patency**.

The patient's post-operative instructions included the administration of a prophylactic dose of low-molecular-weight heparin, limb elevation, and application of compression stockings (class II). During the immediate post-operative and follow-up phase (six months), only mild edema of the leg was marked. The SFV inter-position graft showed good patency (Figure 2) on color duplex ultrasonography.

## Discussion

Pancreatic resection is sometimes combined with reconstruction of the major veins with venous grafts [6]. The vein reconstruction can be applied more frequently than anticipated pre-operatively, since pre-operative imaging can present false estimation of the SMV/PV invasion and CT may not differentiate tumor invasion from inflammatory adhesion [4,7]. The aforementioned examples justify the need for vascular intra-operative consultation, as in our case. From the surgical point of view, it is only when the neck of the pancreas has been divided that the degree of SMV involvement can be assessed to further proceed to SMV/PV resection and reconstruction [8].

Several types of conduits have been utilized for the reconstruction of the SMV, including mostly autogenous vein grafts and, in some cases, synthetic polytetrafluorethylene grafts. The avoidance of the infection risk regarding the pancreatoduodenectomy favors the autogenous conduits. Apart from the commonly used saphenous vein (SV) and SMV, autologous reconstructions with the internal jugular vein, left renal vein, and gonadal veins have also been reported. The SFV provides an excellent size match (7 mm to 12 mm in diameter and 40 to 50 cm in length) for the SMV/PV site compared with the SV [9]. Generally, the SV is preferred for SMV patching, whereas the SFV is preferred as an inter-position conduit. Lee *et al*. [4] suggested performing reconstruction of the SMV/PV with a vein patch when less than one-third of the vessel circumference is involved, whereas an inter-position fashion is the preferred option when there is a greater degree of vessel involvement. Careful preservation of the junction of the profunda femoris vein with the common femoral vein remains a key note for the prevention of excessive venous hypertension.

Immediately post-operatively and after six months, our patient had only mild edema and no discomfort. There seemed to be no significant difference in the measurement of the circumference of the harvested limb compared with the unharvested limb (thigh, proximal calf, mid-calf, and ankle). As long as the SFV harvest does not extend into the popliteal segment and the profunda femoral vein is preserved, the generation of severe venous hypertension and the consequent need for prophylactic fasciotomies is precluded. The minimal mid-term to late-term lower-limb venous morbidity could be attributed to the preservation of collaterals and the low incidence of mild reflux despite the venous outflow obstruction, provided that the venous valves are intact and competent. The deep vein harvest results in venous outflow obstruction. This in turn generates pooling of blood in the periphery and consequent poor apposition of the venous valve leaflets, leading to functional venous reflux, thus underscoring the clinical utility of compression stockings. These pathophysiologic features could explain why SFV harvest is so well tolerated in contrast to the valve damage caused by venous thrombosis.

## Conclusion

The SFV can be an excellent conduit for SMV reconstruction because of its size and availability, good mid-term patency, and low peri-operative and post-operative venous morbidity. Surgeons should be aware of and prepared for the unexpected need to perform venous reconstruction with a SFV conduit. Adherence to technical perfection makes SFV an excellent conduit with minimal morbidity.

## Abbreviations

SFV: superficial femoral vein; SMV: superior mesenteric vein; SMV/PV: superior mesenteric portal vein.

## Consent

Written informed consent was obtained from the patient for publication of this case report and any accompanying images. A copy of the written consent is available for review by the Editor-in-Chief of this journal.

## Competing interests

The authors declare that they have no competing interests.

## Authors' contributions

HG conceived the study concept and design and was involved with the patient's care. EG, JT, and KP were involved in the formation of the study concept and design, patient care, the drafting of the manuscript, and the literature review. CS and HG carried out the operation on the patient. All authors read and approved the final version of the manuscript.
